# NR2B Expression in Rat DRG Is Differentially Regulated Following Peripheral Nerve Injuries That Lead to Transient or Sustained Stimuli-Evoked Hypersensitivity

**DOI:** 10.3389/fnmol.2016.00100

**Published:** 2016-10-18

**Authors:** Monica Norcini, Alexandra Sideris, Samantha M. Adler, Lourdes A. M. Hernandez, Jin Zhang, Thomas J. J. Blanck, Esperanza Recio-Pinto

**Affiliations:** ^1^Department of Anesthesiology, Perioperative Care and Pain Medicine, NYU Langone Medical Center, New York University, New YorkNY, USA; ^2^Department of Neuroscience and Physiology, NYU Langone Medical Center, New York University, New YorkNY, USA; ^3^Department of Biochemistry and Molecular Pharmacology, NYU Langone Medical Center, New York University, New YorkNY, USA

**Keywords:** NR2B, spared nerve injury, neuropathic pain, sensory neurons, DRG, dorsal root ganglia

## Abstract

Following injury, primary sensory neurons undergo changes that drive central sensitization and contribute to the maintenance of persistent hypersensitivity. NR2B expression in the dorsal root ganglia (DRG) has not been previously examined in neuropathic pain models. Here, we investigated if changes in NR2B expression within the DRG are associated with hypersensitivities that result from peripheral nerve injuries. This was done by comparing the NR2B expression in the DRG derived from two modalities of the spared nerve injury (SNI) model, since each variant produces different neuropathic pain phenotypes. Using the electronic von Frey to stimulate the spared and non-spared regions of the hindpaws, we demonstrated that sural-SNI animals develop sustained neuropathic pain in both regions while the tibial-SNI animals recover. NR2B expression was measured at Day 23 and Day 86 post-injury. At Day 23 and 86 post-injury, sural-SNI animals display strong hypersensitivity, whereas tibial-SNI animals display 50 and 100% recovery from post-injury-induced hypersensitivity, respectively. In tibial-SNI at Day 86, but not at Day 23 the perinuclear region of the neuronal somata displayed an increase in NR2B protein. This retention of NR2B protein within the perinuclear region, which will render them non-functional, correlates with the recovery observed in tibial-SNI. In sural-SNI at Day 86, DRG displayed an increase in NR2B mRNA which correlates with the development of sustained hypersensitivity in this model. The increase in NR2B mRNA was not associated with an increase in NR2B protein within the neuronal somata. The latter may result from a decrease in kinesin Kif17, since Kif17 mediates NR2B transport to the soma’s plasma membrane. In both SNIs, microglia/macrophages showed a transient increase in NR2B protein detected at Day 23 but not at Day 86, which correlates with the initial post-injury induced hypersensitivity in both SNIs. In tibial-SNI at Day 86, but not at Day 23, satellite glia cells (SGCs) displayed an increase in NR2B protein. This study is the first to characterize of cell-specific changes in NR2B expression within the DRG following peripheral nerve injury. We discuss how the observed NR2B changes in DRG can contribute to the different neuropathic pain phenotypes displayed by each SNI variant.

## Introduction

In humans with chronic allodynia associated with previous surgical fields and neuromas, altered central processing is not autonomous, and appears to depend on continued peripheral nerve input (Hoffert et al., 1984; Gracely et al., 1992; Gold and Gebhart, 2010). The development of chronic neuropathic pain after surgery is often associated with peripheral nerve injury (Macrae, 2001, 2008). In animal models involving peripheral nerve injuries, increases in excitability and ectopic discharges of injured and closely located uninjured nerve fibers, and from their soma located within the dorsal root ganglia (DRG) alter the stimuli-evoked afferent input that may drive central sensitization and contribute to the maintenance of persistent hypersensitivity (Kingery et al., 1993; Sotgiu and Biella, 1997; Sukhotinsky et al., 2004; Kovalsky et al., 2009; Smith et al., 2013). The *N*-methyl-D-aspartate receptor (NMDAr) is one of many proteins that have been associated with neuropathic pain. The NMDAr is one of the three ligand-gated ion channels activated by glutamate, the major excitatory neurotransmitter in the brain and spinal cord. The NMDAr is a heterotetramer consisting of two NR1 and two NR2 subunits. In neurons, the amount of functional NMDAr is defined by the expression of the four types of NR2 subunits since neurons contain large cytoplasmic pools of NR1 subunit (Brose et al., 1993; Petralia et al., 1994a,b; Hall and Soderling, 1997; Huh and Wenthold, 1999), that are mostly unassociated with NR2 subunits, and hence non-functional (Chazot and Stephenson, 1997; Huh and Wenthold, 1999). Using knockout mice and pharmacological tools, NR2B has been implicated in neuropathic pain (Abe et al., 2005). Treatment with NR2B-selective antagonists decreases pain in various animal models of neuropathic pain (Chizh et al., 2001; Malmberg et al., 2003; Abe et al., 2005; Swartjes et al., 2011). Compared to general NMDAr blockers, specific NR2B antagonists display lower central toxicity both in animal models (sural-SNI, sciatic nerve ligation, spinal nerve ligation, inflammatory pain; Boyce et al., 1999; Swartjes et al., 2011) and in clinical trials (Mony et al., 2009), which reflects the more restricted expression of NR2B in the brain (Watanabe et al., 1993; Farrant et al., 1994). Various studies suggest that the action of NR2B antagonists, in part, is mediated by actions at the spinal cord level. Intrathecal application of an NR2B antagonist reduces mechanical hypersensitivity following L5-spinal nerve ligation (Qu et al., 2009). Intrathecal injections of small interfering RNAs (siRNAs) abolish formalin-induced pain behaviors and decrease the expression of NR2B in the spinal cord (Tan et al., 2005; Zhang et al., 2013). In sural-SNI, oral immunization with anti-NR2B decreases mechanical hypersensitivity and NR2B expression in the spinal cord (but not in the brain; Wang et al., 2007). In rats, intrathecal injections reach both the spinal cord and the closely located DRG (Butler et al., 2005; Wang et al., 2005). Hence, intrathecal injections of NR2B blockers and of siRNAs targeting the NR2B, and oral immunization with anti-NR2B would be expected to reach and affect not only the spinal cord but also the closely located DRG. The observed therapeutic benefits of these treatments could therefore also involve their actions in the DRG. Within the DRG, the primary sensory neurons (Marvizon et al., 2002; Castillo et al., 2013) and their surrounding satellite glia cells (Castillo et al., 2013) express functional NMDAr. Moreover, several lines of evidence indicate that there is glutamate release within the sensory ganglia (Battaglia and Rustioni, 1988; Brumovsky et al., 2007). By using the sniffer patch technique it has been shown that electrical stimulation appears to elicit glutamate release from the somata of DRG neurons (Gu et al., 2010); and reducing glutamine synthase in the trigeminal ganglion produces analgesia in the formalin test (Jasmin et al., 2010). In addition, peripheral Schwann cells store and release glutamate (Parpura et al., 1995; Jeftinija and Jeftinija, 1998). These observations indicate that there is glutamate release in the sensory ganglia and peripheral nerves that contributes to the excitability of peripheral sensory neurons. Since NR2B is the predominant NR2 subunit within the DRG, in this study we investigated how NR2B changes in the peripheral DRG are related to stimuli-evoked hypersensitivities in rats by using two variants of the spared nerve injury (SNI) model. In contrast to the tibial-SNI variant, sural-SNI produces robust, long lasting mechanical and cold allodynia in the ipsilateral paw (Norcini et al., 2014). We investigated whether sustained mechanical and cold hypersensitivity resulting from the sural-SNI variant is accompanied by an increase in NR2B expression within the DRG.

## Materials and Methods

### Animal Model

Adult male Sprague–Dawley rats (250–400 g) were used following the guidelines approved by the New York University Langone Medical Center Institutional Animal Care and Use Committee. Under isoflurane anesthesia, two different variations of the SNI were performed as previously described (Decosterd and Woolf, 2000; Norcini et al., 2014). In sham controls, the sciatic nerve and its branches were only exposed but not manipulated (Norcini et al., 2014).

### Behavioral Test

Mechanical hypersensitivity was evaluated in individual rats placed in Plexiglas boxes upon an elevated metal grid allowing access to the plantar surface of the hind-paws (Norcini et al., 2014). Rats were “marked” on the top of their tails and were randomly placed into the individual Plexiglas boxes. The investigator doing the measurements could not see the “mark” on the rat’s tail. The individual applying the filament was different from the individual doing the read out on the electronic von Frey apparatus. Mechanical thresholds were measured in two regions of the plantar surfaces of both hindpaws; the middle region which is mostly innervated by the tibial nerve, and the lateral region which is mostly innervated by the sural nerve (Duraku et al., 2012). We also recorded whether the response was accompanied by a paw withdrawal. Mechanical thresholds were obtained by using the electronic von Frey apparatus equipped with a size 15 filament fitted on the 800 g arm (IITC Life Sciences, Inc.). The filament has a uniform flat tip of 0.8 mm in diameter, allowing a consistent stimulus surface area. When the filament contacts the paw, the recording unit displays the amount of pressure applied in grams; this value increases as the pressure being applied is increased. When the animal withdraws the paw the maximum grams value is stored in the recording unit; this value, is referred as the “mechanical threshold.” If at a given applied pressure the filament starts to bend, without a paw withdrawal, no additional pressure is applied and a constant maximum grams value must be maintained for 3 s in order to be recorded as the “mechanical threshold.” Thus, for each animal, maximum thresholds are recorded, regardless of the presence of a paw withdrawal response. Measurements were repeated three times with an interval of about 3–5 min between stimulations, and for each animal the mean value was computed. Mean values per animal were then pooled together according to surgery variant and day of testing.

### Paw Volume Measurements

Paw volumes were measured using a plethysmometer (Trio-3-in-1-Electronic System, IITC Life Science, Woodland Hills, CA, USA; cat# 2888 and 520A) before and after surgery. For each animal three measurements were done for each time point.

### Lumbar DRG Collection

Dorsal root ganglia used for isolation of total RNA and for Western blots were collected as previously described (Norcini et al., 2014). Total RNA was isolated as previously described (Norcini et al., 2014). Reverse transcription of total RNA was done by using the High Capacity cDNA Reverse Transcription Kit (Applied Biosystems, cat#4368814). DRG used for western blot were snap frozen upon their isolation (liquid nitrogen/dry ice) and stored at -80°C.

For immunohistochemistry DRG were collected as follows: while under isoflurane anesthesia, a transcardial perfusion was performed first with 500 ml ice cold 10% sucrose containing Heparin (10 units/mL; Hospira Inc. Lake Forest, IL, USA) and then with 500 ml ice cold 4% paraformaldehyde (PFA) in phosphate-buffered saline (PBS). A ‘Perfusion Two^TM^ Automate pressure Perfusion “apparatus (Leica, Microsystem; cat# 39471005) was used to circulate these solutions. DRG were collected and put into 4% PFA at 4°C overnight. The next day DRG were put into 30% sucrose at 4°C for 1 week. The tissues were then embedded in Tissue-Tek OCT (Sakura Finetek Inc., Torrance, CA, USA; cat# 25608930), fast frozen with dry ice and stored at -80°C. Thin sections (18 μm) were cut with a cryostat (Leica Microsystem; Model # CM3050-S).

### Immunohistochemistry

For each animal 3–4 sections from different regions within the DRG were used. The sections were surrounded with a PAP-PEN (Scientific Device Laboratory, Des Plaines, IL USA; ca# 9804) and solutions were added directly on the sections. Sections were rinsed five times with PBS and then incubated with blocking/permeabilization solution [1% BSA, 5% serum; Normal goat serum (NGS) or Normal Donkey serum (NDS)] and 0.4% Triton X-100) for 90 min at room temperature (RT). The sections were incubated overnight (at 4°C) with the following primary antibodies: Anti-NMDA𝜀2 Goat Polyclonal (1:100, Santa Cruz Biotechnology, cat# sc-1469); Anti-Glutamine Synthetase clone GS-6 Mouse Monoclonal (1:200, Millipore, cat#MAB302); or with Iba-1 Rabbit Polyclonal (1:500, Wako, cat#019-19741) dissolved in PBS containing 1% NDS or NGS, 1% BSA, and 0.4% Triton X-100. When using the anti- NMDA𝜀2, the day after, the sections were incubated with the primary antibody for one additional hour at RT. The sections were rinsed five times with 0.25% BSA, 0.02% Triton X-100 in PBS, and then incubated at RT for 90 min with the corresponding secondary antibodies (Invitrogen, Molecular Probes): Donkey Anti-Goat Alexa Fluor 456 1:1000 (# A11036), Goat Anti-Rabbit Alexa Fluor 488 1:1000 (# A11034) and Goat Anti-Mouse Alexa Fluor 456 1:1000 (# A11030). The secondary antibodies were diluted in 1% serum (NDS, NGS, or NHS), 1% BSA, and 0.02% Triton X-100 in PBS. Sections were washed three times with 0.1% Triton X-100 in PBS and two times with PBS. Then they were incubated with the Nissl stain (1:100, Invitrogen, # N21480) according to the manufacturer’s protocol. When we performed the triple immunostaining we incubated the two primary antibodies for two consecutive nights (anti-NR2B was always the second one). The Negative sections were incubated only with 2% of the appropriate Serum or with IgG Goat 1:100 (Jackson ImmunoResearch, #005-000-003, working solution 0.2 mg/ml), IgG Rabbit 1:500 (Jackson ImmunoResearch, #011-000-003, working solution of 0.3 mg/ml) and IgG Mouse 1:200 (Jackson ImmunoResearch, #015-000-003, working solution of 0.25 mg/ml) instead of the corresponding Primary Antibody. The IgG working solutions were prepared to have the same concentration of the working solutions of the corresponding primary antibody. An additional negative control consisted in the simultaneous incubation with anti-NR2B and its blocking peptide (BP 1 μg/0.1 ml; NR2B BP cat#sc-1469-P, Santa Cruz Biotechnology). Prior to their addition to the DRG section, the anti-NR2B and BP were premixed (for 2 h at RT with agitation). Coverslips were mounted using Aqua Poly/Mount (Polysciences Inc.; cat# 18606).

### Analysis of Images

Images were captured and analyzed with a Zeiss Axiovert 200 (Carl Zeiss, Germany) inverted microscope equipped with fluorescence and Nomarski optics, ApoTome (for optical sections), using an Axiocam camera (Zeiss) and AxioVision Software version 4.6.3 (Carl Zeiss imaging systems, Thornwood, NY, USA). The pictures of the entire section were taken with a 40× objective.

### Measuring NR2B Intensity Labeling

**FIGURE 1:**
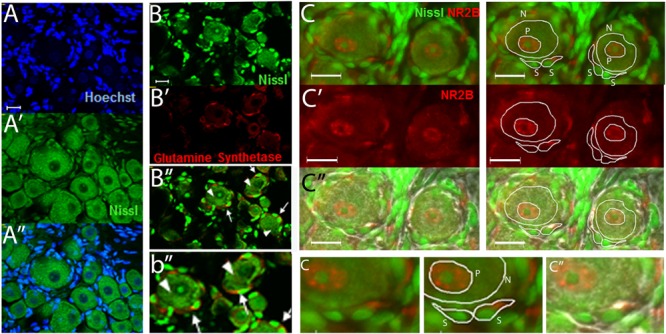
**The use of Nissl staining for the identification and selection of neuronal and satellite glia cell (SGC) areas that do not overlap with each other. (A–A”)** DRG section stained with Nissl (green) and with the nuclear stain Hoechst (blue). Nissl staining labels the neuronal cell bodies and the nuclei of SGCs. The latter can be seen by the colocalization of the nuclear staining (Hoechst, blue) and the Nissl staining (green). **(B–B”)** DRG section immunostained with anti-glutamine synthase (red) and stained with Nissl (green). “**b**” is an amplification of an area in **B**.” Some neurons (arrow heads) and SGCs (arrows) are indicated. **(C–C”)** DRG section immunostained with anti-NR2B (red) and stained with Nissl (green). White lines show examples of the selection of neuronal “N,” perinuclear “P,” and SGC “S” regions used to measure the level of NR2B labeling. In **(c”)**, the light image was also overlapped. Small “**c**” is an amplification of an area in the above **(C, C”)** pictures. Optical sections (0.86 um thick, Apotome) **(A–B)** and regular pictures **(C)**. Magnification 40× objective. Scale bars 20 μm.

Regions of neuronal somata and satellite glial cells (SGCs) that did not overlap with each other were located by using Nissl staining as previously described (Castillo et al., 2013) and were used to measure the level of NR2B labeling (**Figure 1**). The cells that closely surround the neuronal somata have been identified as SGCs by various glial markers such as with anti-glutamine synthetase (Weick et al., 2003; Hanani, 2005; Castillo et al., 2013) (**Figures 1B–B”,b”**). Within the neuronal soma NR2B labeling is highest at the perinuclear region (**Figure 1C**, labeled “P”) as we previously reported (Castillo et al., 2013). Hence, we also measured the level of NR2B labeling in the perinuclear region (**Figure 1**).

**FIGURE 2:**
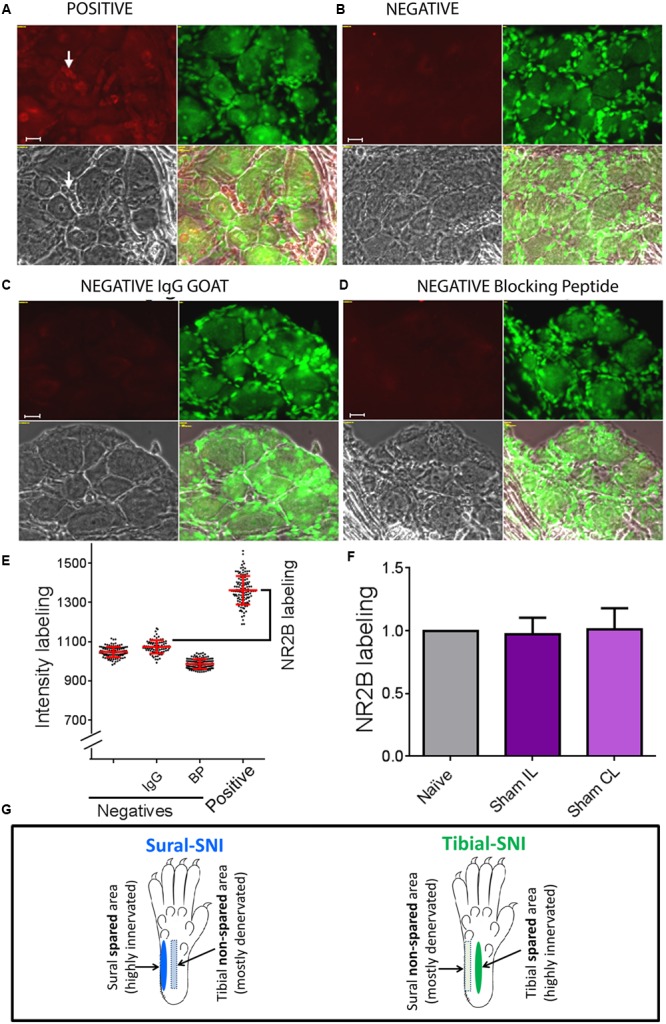
**Determination of specific NR2B labeling.** To determine the specific NR2B labeling, DRG sections were incubated with anti-NR2B **(A)** and three negative controls were used, one in which the primary antibody (anti-NR2B) was omitted **(B)**, another in which the primary antibody was replaced with goat IgG **(C)**, and the third one in which the primary antibody was pre-incubated with blocking peptide **(D)**. **(A–D)** Show four panels: top left corresponds to the red signal: NR2B-labeling + background **(A)** or only background **(B-D)**. Top right: Nissl staining. Bottom left: light picture. Bottom right: overlap of the three panels. **(E)** Shows intensity labeling in the red channel for the four conditions. Each dot represents a measurement from a single soma, red lines indicate the mean ± SD. Based on this result we decided to use the negative IgG goat group to set the background value, which consisted in the average value plus one standard deviation. Specific NR2B intensity labeling (NR2B labeling) was defined as: mean value of the “Positive group” minus the mean value + standard deviation of the “negative IgG group” (indicated with the bracket). **(F)** Shows that the “NR2B labeling” for the neuronal soma was the same for Naïve and Sham rats (mean ± SEM, normalized to the Naïve values). No significant difference was found between any of the groups (One-way ANOVA). The same was observed for SGCs and perinuclear areas between Naïve and Sham rats (not shown). **(G)** The sural and tibial nerves innervate the lateral and middle areas, respectively. In sural-spared nerve injury (SNI) the sural area is spared and the tibial-area in non-spared. In tibial-SNI the tibial area is spared and the sural area in non-spared. Rectangle indicate the non-spared regions and ovals the spared regions for each of the SNI variants.

**Figure 2**, shows images of sections with positive staining (**Figure 2A**) and for three negative controls used for immunostaining with anti-NR2B (**Figures 2B–C**) as described under immunohistochemistry. In **Figure 2E**, each dot, represents the measurement of a single neuronal soma and shows that the distribution of intensity labeling for the three negative controls was similar. For each animal, the “intensity of NR2B labeling” was obtained by subtracting the mean value plus standard deviation of the intensity labeling in the “IgG negative group” from the mean value of the intensity labeling in the “positive group” (**Figure 2E**, black bracket). We found that the level of NR2B intensity labeling in the neuronal soma was not changed in Sham operated animals, as compared to that observed in naïve animals (**Figure 2F**). Hence, for control values we pooled the data from naïve and sham animals.

### Western Blot

Dorsal root ganglia were placed in a 1:30 proportion into a RIPA Lysis solution (Millipore, Billerica, MA, USA, # 20-188) containing 1% Igepal (Sigma-Aldrich, CA-630), 0.1% SDS, 1:100 Protease Inhibitor Cocktail (Rockford, IL, USA, cat#78410) and 1:100 of Phosphatase Inhibitor Cocktail (Pierce, Thermo Fisher Scientific Inc., and cat# 78420). The tissue was homogenized using an automatic cordless motor pestle mixer (cat#K749540-000, Kimble Chase Kontes). The suspension was left on ice for 30 min, vortexed (5–7 s) and centrifuged at 13,000 rpm for 15 min at 4°C. The supernatant was collected, aliquoted and stored at -80°C until used. Protein concentration was determined by the Lowry Assay (Sigma-Aldrich). The sample volume containing 10–20 μg of protein was brought to a total volume of 20 μl by adding RIPA Enriched containing Protease Inhibitor Cocktail (1:100) and 10 μl of loading 2x tris buffer (1.25 ml of 0.5M Tris HCl, pH 6.8, 2.5 ml Glycerol, 2.0 ml of 10% SDS, 0.2 ml of 0.5% Bromophenol blue and 3.55 ml water). Samples were processed and electrophoresis was run as previously described (Castillo et al., 2011). Primary antibodies used: rabbit polyclonal Anti-NR2B subunit (1:2000; Abcam Inc., Cambridge, MA, USA; cat#ab14400) and goat polyclonal Anti-β-Actin (1:5000, Abcam; cat# ab8229); and secondary antibodies used: Goat anti-Rabbit (1:1000, cat# sc-2004) or Rabbit anti-Goat (1:10,000, cat# sc-2768; Santa Cruz Biotechnology).

### Preparation of the cDNA from Total RNA

Total RNA extraction from DRGs was done as previously described (Norcini et al., 2014). The quality of the total RNA samples was determined with the 2100 Bioanalyzer (Agilent Technologies). The total RNA samples had an RNA integrity number (RIN) between 8.4 and 9.5 (the maximum RIN value is 10). The total RNA concentration was determined using the NanoDrop^®^ ND-1000 Spectrophotometer (Thermo Fisher Scientific), and ranged between 90.5 and 327.6 ng/μl. We converted total RNA into cDNA by using the High Capacity cDNA Reverse Transcription Kit (Applied Biosystems, cat#4368814). We performed the RT step in an Eppendorf ep gradient S Mastercycler with a ramp speed of 6°C/s. The thermal-cycling conditions used were the ones suggested from the manufactures.

### RT-PCR for mRNA

The expression of the messenger RNAs for the NMDA receptor subunits, the kinesin family member 17 (Kif17), and the kinesin family member 5b (Kif5b) was quantified by real-time qRT-PCR and it was performed using a CFX96 Touch^TM^ apparatus from BIO-RAD and Multiplate^TM^ Low-Profile 96-Well Unskirted PCR Plates (BIO-RAD, cat#MLL9601). TaqMan Gene Expression Master Mix (Applied Biosystems, #4369016) was used according to the protocol. The TaqMan Gene Expression Assays (TaqMan Assays from Applied Biosystems, Life Technologies, Carlsbad, CA, USA, #4331182) used are the following: Grin1: Glutamate Receptor, Ionotropic, *N*-Methyl D-Aspartate 1, ID#Rn01436038_m1; Grin2A: Glutamate Receptor, Ionotropic, *N*-Methyl D-Aspartate 2A, ID#Rn00561341_m1; Grin2B: Glutamate Receptor, Ionotropic, *N*-Methyl D-Aspartate 2B, ID#Rn00680474_m1; Grin2C: Glutamate Receptor, Ionotropic, *N*-Methyl D-Aspartate 2C, ID#Rn00561359_m1; Grin2D: Glutamate Receptor, Ionotropic, *N*-Methyl D-Aspartate 2D, ID#Rn00575638_m1 and GAPDH: glyceraldehyde-3-phosphate dehydrogenase, ID#Rn01775763_g1. Expression Assays (TaqMan Assay from Applied Biosystems, Life Technologies, Carlsbad, CA, USA, #4351372) was used for Kif17: ID#Rn01515647_m1; and the (TaqMan Assay from Applied Biosystems, Life Technologies, Carlsbad, CA, USA, #4331182) was used for Kif5b: ID#Rn01538432. For a given sample, all reactions were run in duplicate. Background controls consisted of replacing the cDNA with water. Forty five cycles of amplification were done. Data (*C*_t_ values were analyzed using a comparative ΔΔ*C*_t_ method (Schmittgen and Livak, 2008). GAPDH was used as the endogenous control to obtain the Δ*C*_t_ value for each of the probes within each DRG and spinal cord. GAPDH has been validated as a stable normalization gene for qRT-PCR when using DRG and spinal cord samples derived from the SNI model (Piller et al., 2013). For each probe the ΔΔ*C*_t_ was obtained by using the Δ*C*_t_ experimental value (sural-SNI or tibial-SNI DRG) minus the Δ*C*_t_ control value (Sham DRG). Then the fold change (2^-ΔΔ^*^C^*^t^) was calculated.

### Statistical Analyses

Statistics were done by using GraphPad Prism vs. 6.07. One-way ANOVA was used when comparing more than two groups, followed by Bonferroni’s multiple comparison test or by Tukey’s multiple comparison test. Two-way ANOVA was used when comparing two or more groups over time. Two-way ANOVA Ordinary was used when there was not an equal number of data for each of the days. Two-way ANOVA Matching Stacked was used when there was an equal number of data for each of the days. Two-way ANOVA was followed by Bonferroni’s Multiple comparison.

## Results

### Both Tibial (Middle) and Sural (Lateral) Regions of the Hindpaw Develop Mechanical Hypersensitivity

**FIGURE 3:**
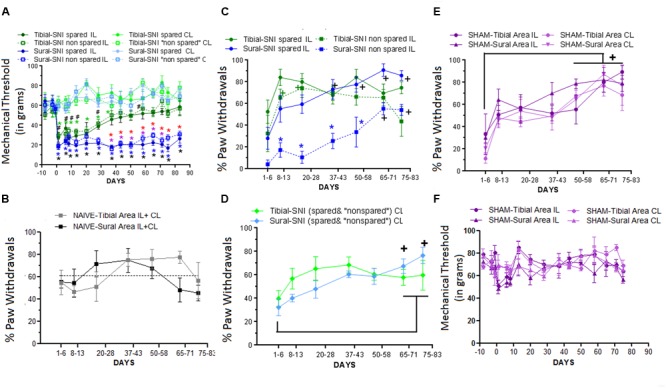
**Differences in Mechanical Thresholds and Percentage of Paw Withdrawals between spared and non-spared regions in the tibial-SNI and sural-SNI models. (A)** Mechanical Thresholds measurements in the ipsilatereal (IL) and contralateral (CL) paws in both the spared and non-spared regions of sural-SNI and tibial-SNI rats. No significant difference was found between the spared vs. non-spared regions in the IL paws for the tibial-SNI, neither for the sural-SNI. Significant different: symbols in black: non-spared-IL vs. non-spared-CL for a given SNI (# tibial-SNI, ^∗^sural-SNI). 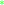
(green): spared-IL vs. spared-CL regions in tibial-SNI. 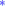
(blue): spared-IL vs. spared-CL regions in sural-SNI. 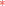
(red): spared-IL tibial-SNI vs. spared-IL sural SNI. 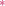
(purple): non-spared-IL tibial-SNI vs. non-spared-IL sural-SNI. For all the groups *n* = 6 rats for most days, except for Days 20, 71, and 75 where *n* = 3 rats, and Day 83 where *n* = 4 rats. One exception is for tibial-SNI non-spared (CL and IL) that the *n* = 3 rats on Day 1. **(B)** Percentage of Paw Withdrawals in the Tibial-area (IL and CL) and Sural-area (IL and CL) in naïve rats. No significant difference was found when comparing between groups at a given day, or within a group between 1–6 Days vs. later days; *n* = 3 rats. **(C)** Percentage of Paw Withdrawals in the IL paws of sural-SNI and tibial-SNI. Comparison between groups at a given day: 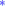
(blue): spared-sural-SNI vs. non-spared sural-SNI. No significant difference was found between spared-tibial-SNI vs. non-spared-tibial-SNI; or between spared-tibial-SNI vs. spared-sural-SNI. Within a group between 1–6 Days vs. later days: **+** (black): sural-SNI (spared and non-spared); 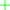
(green) tibial-SNI (spared and non-spared). *n* = 6 rats (same animals as in **A**). **(D)** Percentage of Paw Withdrawals in the CL paws of sural-SNI and tibial-SNI in the CL. For a given day, no significant difference between the sural-SNI-CL and tibial-SNI-CL regions. Within a group between 1–6 Days vs. later days: no significance difference in tibial-SNI; **+** (black): sural-SNI. **(E)** Percentage of Paw Withdrawals in the tibial-area (IL and CL) and sural-area (IL and CL) in Sham rats. For a given day, no significant difference was found between the groups. For a given group, significant difference was found between 1–6 Days vs.≥50 Days for three of the groups. The fourth group, the tibial-Area-IL, significant difference was found between 1–6 Days vs.≥65 Days. *n* = 4 rats; **(F)** Mechanical Threshold measurements in the IL and CL paws in both Sural and Tibial areas. No significant differences were found; between groups within a given day. Two-way ANOVA Ordinary **(A)** and Two-way ANOVA Matching Stacked **(B–F)**; followed by Bonferroni’s Multiple comparison (all symbols: *p* < 0.05). Values are mean ± SEM.

Responses to mechanical stimulation to the middle (tibial) and lateral (sural) regions of the hindpaws were compared between the two SNI variants (**Figure 2G**). The middle region is mostly innervated by the tibial nerve, and the lateral region is mostly innervated by the sural nerve. The corresponding spared and non-spared regions for each SNI are shown in **Figure 2G**. The onset of mechanical hypersensitivity and its highest level, corresponding to the lowest mechanical threshold value, was detected on Day 1 post-surgery and had a similar magnitude in the spared regions of both SNI variants (**Figure 3A**). However, while the sural-SNI animals maintained a low mechanical threshold throughout the entire observation period (**Figure 3A**, dark blue) the tibial-SNI animals started to recover from mechanical hypersensitivity, as indicated by the increase in their mechanical threshold value, at about 2 weeks and displayed almost full recovery by Day 40 post-surgery (**Figure 3A**, dark green), as we previously reported (Norcini et al., 2014). We now report that, within each SNI variant, the mechanical thresholds were the same for the corresponding “spared” and “non-spared” regions (**Figure 3A**, solid lines vs. dashed lines-dark green and dark blue). However, the spared and non-spared regions displayed qualitative differences in their mechanical response as could be assessed by recording whether the animals displayed a paw withdrawal. We further characterized the responses to this mechanical stimulus by calculating the percentage of responses that were accompanied by a paw withdrawal (% Paw Withdrawals).

In naïve animals the “% Paw Withdrawals” showed no significant differences among the various times points (**Figure 3B**). The average “% Paw Withdrawals” in naïve rats was ∼61% and comparable for the sural and tibial paw regions (sural region: 60 ± 7%; tibial region: 62 ± 7%, mean ± SEM, *n* = 3 rats; for each animal the value was calculated by averaging all their values measured over time). In the sural-SNI rats, the non-spared region (in this case corresponding to the middle tibial area) displayed a significant decrease in the “% Paw Withdrawals” up to Day 58 post-surgery (**Figure 3C**). This decrease disappeared with time such that by Day 66 onward, there was no significant difference between the spared and non-spared regions in sural-SNI animals. In contrast, such difference was not observed in the tibial-SNI model, in which the “% Paw Withdrawals” was comparable in the spared and non-spared regions (**Figure 3C**). Although within a SNI variant, both the non-spared and spared areas displayed the same decrease in mechanical threshold, qualitatively in the sural-SNI, but not in the tibial-SNI, there were differences between the two regions such that the non-spared area showed a decrease “% Paw Withdrawals” during the first 50 days following the surgery. This difference in “% Paw Withdrawals” disappeared over time while the level of “Mechanical Threshold” remained low and constant.

Interestingly, in each paw region stimulated (middle and lateral) during the first week there was a tendency for a decrease in the “% Paw Withdrawals,” in both the ipsilatereal (IL; **Figure 3C**) and contralateral (CL; **Figure 3D**) paws regardless of SNI. This decrease was also observed in the Sham group (**Figure 3E**), but not in the naïve group (**Figure 3B**). However, in Sham animals, the mechanical threshold was not affected (**Figure 3F**). Hence, the decrease in % Paw Withdrawals within the first week in part results from surgical procedures distinct to nerve injury.

As indicated above, we found no correlation between the “% Paw Withdrawals” and the “mechanical threshold value”; hence both measurements appear to be assessing different aspects of mechanical sensation. The “Mechanical Threshold” measures mechanical sensitivity and the percentage of paw withdrawals likely measures a qualitative difference in the mechanical sensation, that appears to be related to the level of innervation (see “Discussion” section).

In order to investigate whether the two SNI variants displayed differences in paw inflammation, potentially induced by the degeneration of transected fibers, we measured the volume change of the paw. We found that there were no detectable differences in the paw volumes between sham and either SNI group, or between the two SNI groups, in either the IL or the CL paws (**Supplementary Figure S1**). Hence, both SNI variants do not result in detectable levels of paw edema.

### NR2B Expression Is Differentially Regulated in Sural-SNI and Tibial-SNI

Both SNI variants induce strong mechanical hypersensitivity, but only in the sural-SNI does this behavior persist for over 3 months. Moreover, only the sural-SNI rats develop cold hypersensitivity (Decosterd and Woolf, 2000; Norcini et al., 2014). In the SNI models, the hypersensitivity that develops in the rat hind paw from transecting some or all of the sciatic nerve branches is evoked by stimulation of the nerve terminals of the spared sciatic nerve branches and from the saphenous nerve that become hyperexcitable as a result of interactions with the injured sciatic nerve fibers (Kingery and Vallin, 1989; Kingery et al., 1993; Ro and Jacobs, 1993; Attal et al., 1994; Tal and Bennett, 1994; Guilbaud et al., 1995; Sotgiu and Biella, 1997; Campbell, 2001; Smith et al., 2013). Moreover, these fibers (spared sciatic fibers and saphenous fibers) not only become hyperexcitable but they also sprout and reinnervate the areas that were denervated following transection of some of sciatic nerve fibers. In rat, the sensory neurons of the saphenous nerve are located mostly in the L3-DRG and those of the sciatic nerve mostly in the L4- and L5-DRG. Based on the composition of afferent fibers within each of the sciatic nerve branches (Swett et al., 1991), L4 DRG contains the highest number and percentage of injured primary sensory neurons following sural-SNI (**Table 1**). Hence, we measured NR2B expression in L3-DRG and L4-DRG. We selected Day 23 and Day 86 post-injury to measure NR2B labeling because significant behavioral differences between the two models are evident. At Day 23, while there is 50% recovery in mechanical thresholds, and no cold allodynia in tibial-SNI rats, sural animals display strong mechanical and cold hypersensitivity. At Day 86, tibial-SNI rats fully recover, while thermal and mechanical hypersensitivities persist in the sural-SNI rats (**Figure 3A**). Thus, Day 23 and Day 86 measurements of NR2B expression provide information regarding how changes in this subunit is correlated with recovery or persistent pain.

**Table 1:** The number of sensory fibers within each of the sciatic nerve branches (peroneal, tibial, sural) that contributes to each of the Dorsal root ganglias (DRGs; L3, L4, and L5) was taken from (Swett et al., 1991).

	Sural-SNI	Tibial-SNI	Sensory neurons^1^	
	# (%) Injured sensory neurons	# (%) Injured sensory neurons	#neurons (% of a given sciatic nerve branch)^1^	#sensory neurons in DRG mean^2,3^
L3-DRG^1^	113(∼0.8%)	65 (∼0.5%)	65 (2.4% of peroneal)
			48 (1.0% of tibial)
			0 (0% of sural)
L4-DRG^1^	5,367 (43%)	2,208 (18%)	2133 (79% of peroneal)	12,496 (12,000–12,991)
			3234 (68.1% of tibial)
			75 (4.5% of sural)
L5-DRG^1^	1,967 (13%)	2065 (13%)	501 (18.6% of peroneal)	15,321 (15,000–15642)
			1466 (30.9% of tibial)
			1564 (93.4% of sural)
#/type neurons injured in each SNI	7,447 sensory (68%)^1^	4,338 sensory (39%)^1^
	1,614 motor	700 motor		
	13,200 sensory^3^	8,200 sensory^3^
	1,600 motor	600 motor
	4,800 sympathetic	2,600 sympathetic

*Sprague–Dawley rats: ^1^(Swett et al., 1991) and ^2^(McKay Hart et al., 2002). Wistar rats: ^3^(Schmalbruch, 1986). To estimate the percentage of injured sensory neurons within each DRG we used the total number of sensory neurons reported for the L4-DRG and L5-DRG in two studies (Schmalbruch, 1987; McKay Hart et al., 2002). Since no values were available for L3-DRG we used an average value of those reported for the L4-L5 DRG (13,908). Although those two studies used different rat species (Sprague–Dawley and Wistar rats), they found a comparable number of sensory neurons within the DRG and of motor fibers within the various sciatic nerve branches. However, the number of sensory fibers within each of the sciatic nerve branches was very different, being almost double in Wistar rats than in Sprague–Dawley rats. We are showing the values from the Schmalbruch study, since it also provides us with the number of sympathetic fibers within each of the sciatic nerve branches, which allowed us to provide an estimate of the number of sympathetic fibers that would be expected to be injured in both spared nerve injurys (SNIs). This was done to help us in the discussion.*

**FIGURE 4:**
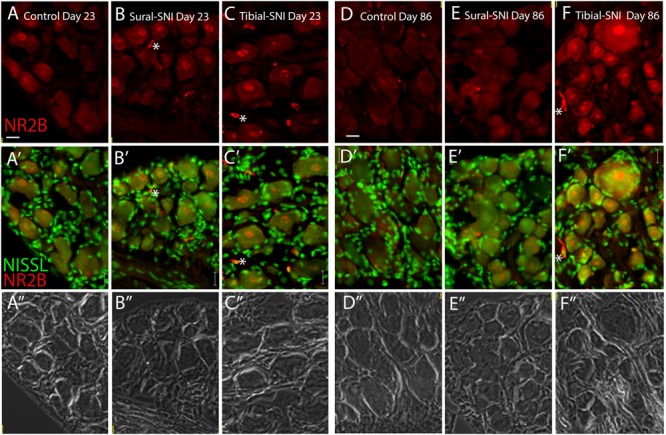
**NR2B intensity labeling in L3-DRG at Day 23 and Day 86 following surgery.** L3 DRG sections were immunostained with anti-NR2B (red) and stained with Nissl (green) at Day 23 for control **(A)**, Sural-SNI **(B)** and Tibial-SNI **(C)** and at Day 86 for control **(D)**, Sural-SNI **(E)**, and Tibial-SNI **(F)**. Top panels only show NR2B intensity labeling (background has been substracted) **(A–F)**; middle panels show NR2B intensity labeling (red) and Nissl staining (green) **(A’–F’)**; and bottom panels shows the corresponding light pictures **(A”–F”)**. Magnification 40× objective. Scale bars: 20 μm.

**FIGURE 5:**
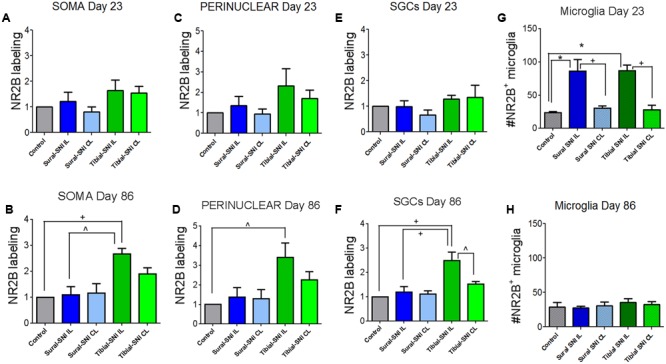
**Quantification of NR2B labeling in various cells in the L3-DRG at Day 23 and Day 86 following surgery.** NR2B labeling in the neuronal soma (SOMA) **(A,B)**, the perinuclear area of the neuronal soma **(E,C)**, in SGC **(E,F)** and in microglia/macrophages **(G,H)** at Day 23 **(A,C,E,G)** and Day 86 **(B,D,F,H)** following surgery. For Day 23 **(A,C,E,G)**: the control group *n* = 6 rats (three sham, three naïve). For each control rat measurements were done in the IL and CL L3-DRG. Measurements were done in on average in 198 somas/rat **(A)**; on average in 198 perinuclear areas/rat **(C)**; on average in 120 SGCs/rat **(E)**. For the other four groups (Sural-SNI-IL, Sural-SNI-CL, Tibial-SNI-L, and Tibial-SNI-CL) *n* = 3 rats. The “average number of somas/rat-area” measured were 116, 101, 111, 109 for Sural-SNI-IL, Sural-SNI CL, Tibial-SNI IL, and Tibial-SNI CL, respectively. The “average number of perinuclear areas/rat-area” measured were 109, 100, 102. 92 for Sural-SNI-IL, Sural-SNI CL, Tibial-SNI IL, and Tibial-SNI CL, respectively. The “average number of SGCs/rat-area” measured were 72, 62, 66, 60 for Sural-SNI-IL, Sural-SNI CL, Tibial-SNI L, and Tibial-SNI CL, respectively. For Day 86 **(B,D,F,H)**: the control group *n* = 4 rats (two sham, two naïve). For each control rat measurements were done in the IL and CL L3-DRG. Measurements were done in on average in 256 somas/rat **(B)**; on average in 257 perinuclear areas/rat **(D)**; on average in 360 SGCs/rat **(F)**. For the other four groups (Sural-SNI-IL, Sural-SNI-CL, Tibial-SNI-L, and Tibial-SNI-CL) *n* = 3 rats. The “average number of somas/rat” measured were 148, 124,122, and 166 for Sural-SNI-IL, Sural-SNI CL, Tibial-SNI IL, and Tibial-SNI CL, respectively. The “average number of perinuclear areas/rat” measured were 148, 137,143, and 190 for Sural-SNI-IL, Sural-SNI CL, Tibial-SNI IL, and Tibial-SNI CL, respectively. The “average number of SGCs/rat” measured were 270, 154, 209, and 256 for Sural-SNI-IL, Sural-SNI CL, Tibial-SNI L, and Tibial-SNI CL, respectively. **(G,H)** Number of microglia/macrophages that were NR2B positive/324,000 μm^2^. The *n* = 6 and 4 rats for Day 23 and Day 86, respectively; and *n* = 3 rats for all the other groups. In all panels: mean + sem. For **(A–F)**, the data was normalized to control values. One-way ANOVA, with Tukey’s multiple comparison test. ^∗^*p* < 0.001, +*p* < 0.01, ˆ*p* < 0.05.

In L3-DRG, at Day 23 post-surgery there were no significant differences in the level of NR2B labeling (in red) in the neuronal somata and SGCs between either tibial-SNI or sural-SNI and the control group (**Figures 4A–C** and **5A,C,E**). In contrast, at Day 86 post-surgery, there was an increase in the intensity of NR2B labeling in the neuronal somata and SGCs from tibial-SNI but not from sural-SNI (**Figures 4D–F** and **5B,F**). The increase in NR2B labeling in the neuronal somata is primarily found within the perinuclear region of the neuron (**Figures 5B** vs. **5D**).

**FIGURE 6:**
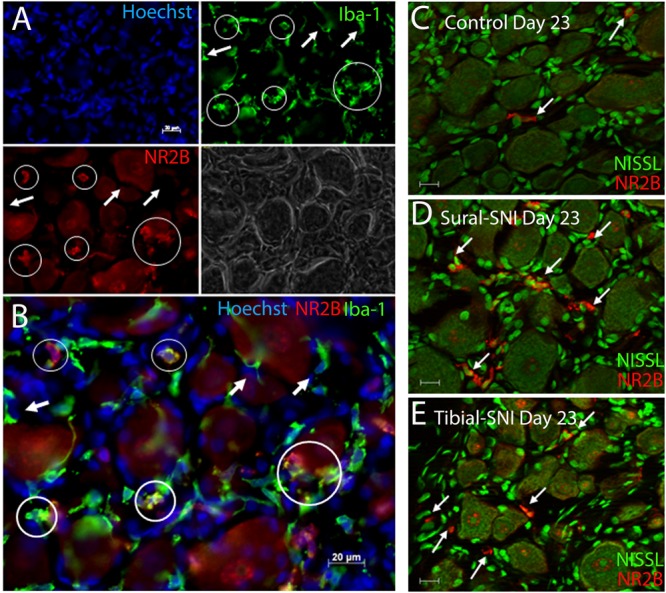
**Identification of microglia/macrophages. (A)** DRG section immunostained with anti-Iba-1 (green) a microglia/macrophage marker and stained with the nuclear stain Hoechst (blue). Both Iba-1+/NR2B+ cells (circles) and Iba-1+/NR2B- cells (arrows) were detected. The corresponding light picture is also shown. **(B)** shows the overlap of the color images in **(A)**. DRG sections immunostained with anti-NR2B (red) and stained with Nissl (green) from a control **(C)** Sural-SNI **(D)** and Tibial-SNI **(E)** L3-DRG at Day 23 post-surgery. **(A–B)** Conventional pictures and **(C–E)** optical sections (0.86 μm thick, Apotome). Magnification 40× objective. Scale bars: 20 μm.

The presence of small cells with intense NR2B labeling were also observed, and were morphologically distinct from SGCs and neurons (**Figure 4**, some indicated with “∗”). Co-labeling with anti-Iba-1 (green) confirmed that these NR2B-positive cells (red) are microglia/macrophages (**Figures 6A,B**, green, and circles); however, not all Iba-1 positive cells expressed NR2B (arrows; **Figures 6A,B**). At Day 23, an increase in the number of microglia/macrophage cells that were NR2B-positive were observed in the ipsilateral DRG of sural-SNI and tibial-SNI rats (**Figures 6C–E** and **5G**). This increase was transitory since it became undetectable on Day 86 post-surgery (**Figure 5H**).

**FIGURE 7:**
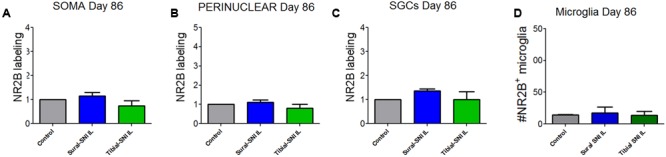
**Quantification of NR2B labeling in various cells in the L4-DRG at Day 86 following surgery.** NR2B labeling in the neuronal soma (SOMA) **(A)**, the perinuclear area of the neuronal soma **(B)**, in SGC **(C)** and in microglia/macrophages **(D)** at Day 86 following surgery. The control group *n* = 5 rats (three naïve and two sham). For each control rat measurements were done in the IL and CL L4-DRG. Measurements were done in on average in 803 somas/rat **(A)**; on average in 564 perinuclear areas/rat **(B)**; on average in 576 SGCs/rat **(C)**. For the other four groups (Sural-SNI-IL, Sural-SNI-CL, Tibial-SNI-L, and Tibial-SNI-CL) *n* = 3 rats. The “average number of somas/rat-area” measured were 294, 558, 358, and 396 for Sural-SNI-IL, Sural-SNI CL, Tibial-SNI IL, and Tibial-SNI CL, respectively. The “average number of perinuclear areas/rat-area” measured were 176, 351, 169, and 181 for Sural-SNI-IL, Sural-SNI CL, Tibial-SNI IL, and Tibial-SNI CL, respectively. The “average number of SGCs/rat-area” measured were 243, 285, 232, 204 for Sural-SNI-IL, Sural-SNI CL, Tibial-SNI L, and Tibial-SNI CL, respectively. **(D)** Number of microglia/macrophages that were NR2B positive/324,000 μm^2^. The *n* = 5 for control and and *n* = 3 rats for all the other groups. In all panels: mean + SEM. For **(A–C)**, the data was normalized to control values. One-way ANOVA.

In contrast to L3-DRG, the neuronal somata and SGCs did not show significant changes in NR2B labeling in L4-DRG at Day 86 post-surgery (**Figure 7**).

Though fluorescence analysis of stained tissue sections demonstrated cell-specific and localized changes of NR2B labeling, significant NR2B differences were not detectable through Western Blot of whole DRG cell lysates of individual L3-DRG and L4-DRG (**Supplementary Figures S2A,B**). In addition to the neuronal somata, the SGCs and microglia, a large proportion of the DRG are comprised of fibers (**Supplementary Figure S2C**). Many of these fibers are also myelinated by Schwann cells, which recently have been shown to also express NR2B (Mantuano et al., 2015). The presence of these various cell types, together with the difference in contribution of the nerve fibers likely complicated the detection of differences in NR2B expression when using Western Blotting.

### Expression of mRNA Encoding the Various NMDA Receptor Subunits at Day 86 Post-surgery

**FIGURE 8:**
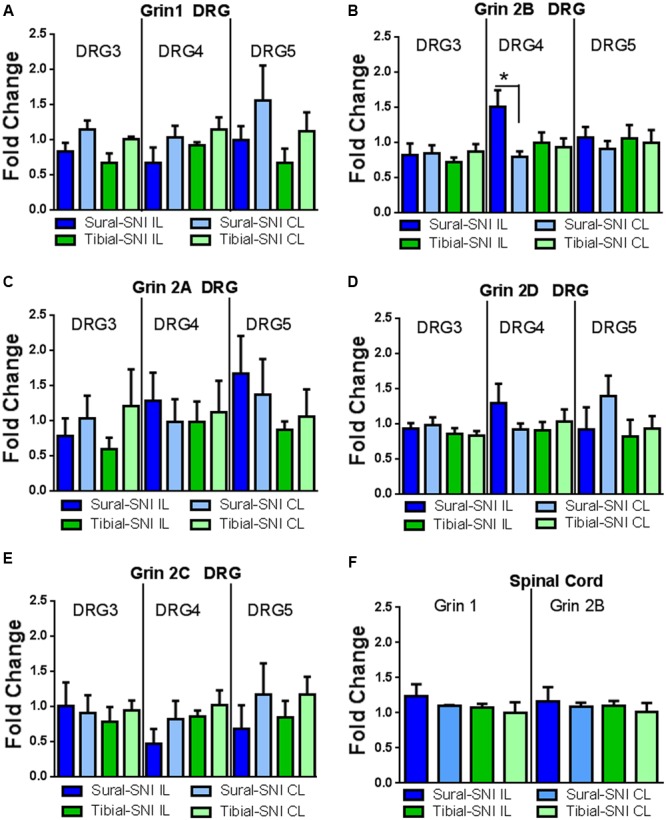
**qPCR for the various NMDAr subunits in DRG and spinal cord derived from sural-SNI and tibial-SNI. (A–E)** For DRG, *n* = 4 rats, except for two groups in Grin1 DRG5 (sural-SNI IL and tibial-SNI CL) *n* = 3. **(F)** For Spinal cord, *n* = 3 rats. For each probe the ΔΔ*C*_t_ was obtained by using the Δ*C*_t_ experimental value (sural-SNI or tibial-SNI DRG) minus the Δ*C*_t_ control value (Sham DRG). Then the fold change (2^-ΔΔ^*^C^*^t^) was calculated. Values are mean + SEM. One-way ANOVA, tukey’s multiple comparison test. ^∗^*p* < 0.05.

Since a significant difference in NR2B labeling was detected only at Day 86 post-surgery between sural-SNI and tibial-SNI, this time point was chosen to also measure mRNA expression for the various NMDA receptor subunits in DRG and in spinal cord tissue by using qPCR. We found that NR2B (Grin 2B) subunit transcripts were up-regulated only in the ipsilateral L4-DRG of sural-SNI (**Figure 8B**). Transcripts for NR1 (Grin1), NR2A (Grin2A), NR2D (Grin2D), NR2C (Grin 2C) subunits were not changed in L3-DRG, L4-DRG, or L5-DRG (**Figure 8**). Moreover, no significant changes were observed in the mRNA for NR1 (Grin1) or NR2B (Grin 2B) in the lumbar spinal cord relative to shams (**Figure 8F**).

### Expression of mRNA Encoding for the Kinesin Family Member 17 (Kif17) and Family Member 5b (Kif5b)

**FIGURE 9:**
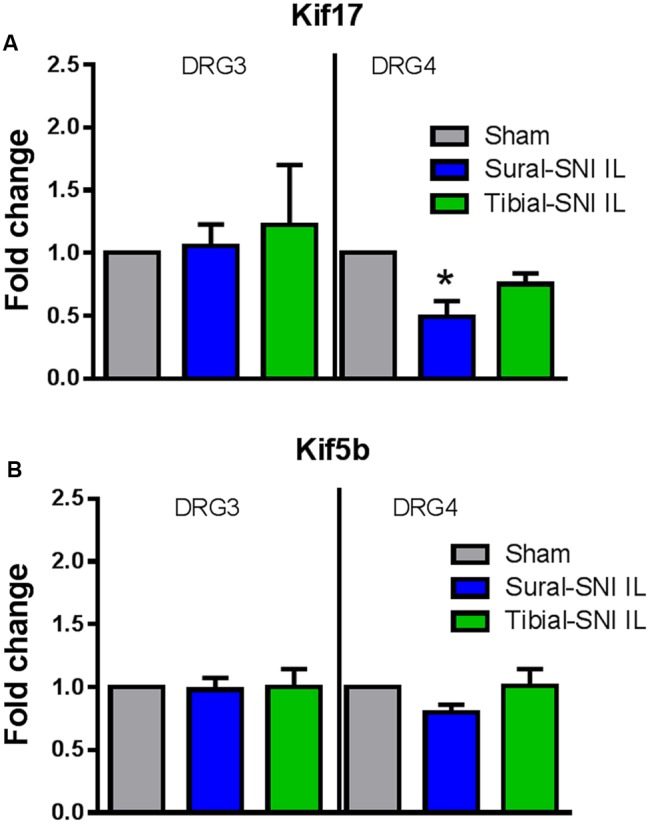
**qPCR for Kif17 and Kif5b in DRG derived from sural-SNI and tibial-SNI.** DRG, *n* = 4 rats. For each probe the ΔΔ*C*_t_ was obtained by using the Δ*C*_t_ experimental value (sural-SNI or tibial-SNI DRG) minus the Δ*C*_t_ control value (Sham DRG). Then the fold change (2^-ΔΔ^*^C^*^t^) was calculated. **(A)** Kif17, **(B)** Kif5b. Values are mean + SEM. One-way ANOVA, tukey’s multiple comparison test. ^∗^*p* < 0.05.

We measured the mRNA expression of the kinesins KIF17 and KIF5B to investigate whether they could contribute to the observed changes in NR2B expression between sural-SNI and tibial-SNI, at Day 86 post-surgery. Kinesins are microtubule associated molecular motors that transport cargos (vesicles, proteins, mRNAs) along microtubules mostly in the anterograde direction (Schnapp, 2003). Of the 47 kinesins identified in mammals, Kif17 is involved in the anterograde transport to dendrites of various proteins including the NR2B subunit (Setou et al., 2000; Wong-Riley and Besharse, 2012); and Kif5b is important in axonal transport (Hirokawa et al., 2010). We found that KIF17 transcripts were down-regulated only in the ipsilateral L4-DRG of sural-SNI (**Figure 9A**), but no change was found in the level of Kif5b transcripts (**Figure 9B**).

## Discussion

We found that the electronic von Frey allows for the detection of mechanical hypersensitivity in the spared and non-spared regions of the hindpaws; however, these regions showed qualitative differences in their responses. We also observed nerve injury-induced changes in NR2B expression in neurons, SGCs and microglia/macrophages of the un-injured L3-DRG and injured L4-DRG. Hence changes in NR2B expression within the DRG appear to affect neuronal and glial function, and may contribute to the neuropathic pain phenotype in SNI.

### Stimuli-Evoked Hypersensitivity

The sural and tibial nerves innervate the lateral and middle regions of the hindpaws, respectively, but there is also spatial overlap (Duraku et al., 2012). Hence, to a different extent, both regions maintain a level of innervation following SNI. With the manual von-Frey, mechanical hypersensitivity is detected only in the sural region following sural-SNI (Duraku et al., 2012, 2013; Nascimento et al., 2015). With the electronic von-Frey, we detected mechanical hypersensitivity in both regions. The manual system uses a set of filaments of varying diameters so as to provide a range of forces (g) (Bradman et al., 2015). With the manual system a mechanical response is detected with thin fibers (0.229–0.254 mm diameter, ∼1.202–1.479 g) in sural-SNI animals; but only with a thick fiber (0.483 mm diameter, ∼15.136 g) in sham animals ^1^ (Wang et al., 2011). The electronic von Frey system used in this study only uses one filament with a 0.8 mm tip diameter, hence a constant area is stimulated and the only variable is the applied pressure. The detection of mechanical hypersensitivity in the highly denervated region may in part reflect the stimulation of a relatively larger area when using the electronic system rather than when using the manual one.

In sural-SNI, the non-spared and spared regions displayed the same decrease in mechanical threshold, while the non-spared region displayed a lower percentage paw withdrawals. However, the “% Paw Withdrawals” slowly increased, and by Day 65 was similar to that of the spared region. This gradual increase in the “% Paw Withdrawals” roughly correlates with the reported time course of reinnervation by sensory fibers (Duraku et al., 2012, 2013). Therefore, in sural-SNI there is a qualitative difference in the mechanical hypersensitivity in these two paw regions, which appears to be related to the level of innervation. In tibial-SNI, the non-spared and spared regions displayed a transient decrease in mechanical threshold, while the “% Paw Withdrawals” was comparable to those in sham animals. Based on the fiber composition of the sciatic nerve (Swett et al., 1991) a lower percentage of afferent fibers are injured following tibial-SNI compared to sural-SNI (39% vs. 68%; **Table 1**). Hence a decrease in “% Paw Withdrawals” requires a high level of sensory denervation.

No correlation was found between the “Mechanical Threshold” and the “% Paw Withdrawals”; hence both measurements assess different aspects of mechanical sensation. While “Mechanical Threshold” measures the mechanical sensitivity, the “% Paw Withdrawals” appears to be related and proportional to the level of sensory innervation. By combining both measurements, one could obtain information regarding the overall increase in mechanical hypersensitivity. In sural-SNI, both the spared and non-spared regions showed a slow continual increase in overall mechanical hypersensitivity, with the spared region showing a highest value and the strongest increase; while in tibial-SNI both regions displayed a transient and comparable increase in overall mechanical hypersensitivity.

Mechanical hypersensitivity displayed by both SNIs appears to primarily involve changes in primary sensory neurons, but it may also involve changes in sympathetic neurons. The number of damaged sympathetic neurons is twice as high in sural-SNI than in tibial-SNI (**Table 1**). In the sural-SNI sympathetic neurons contribute to cold hypersensitivity; but their reported contribution to mechanical hypersensitivity depends on the protocol used for chemical sympathectomy (Pertin et al., 2007; Nascimento et al., 2015).

### DRG NR2B Expression

We used the different neuropathic pain phenotype between the sural-SNI and tibial-SNI to investigate whether changes in NR2B expression within the DRG correlated with the development of sustained hypersensitivity following a sciatic nerve injury. We selected Day 23 and Day 86 to measure NR2B labeling, because in tibial-SNI at Day 23 there is 50% recovery and at Day 86 there is full recovery in injury-induced mechanical hypersensitivity, while sural-SNI displays strong mechanical hypersensitivity at both time points.

At Day 86 an increase in NR2B mRNA was observed only in sural-SNI; hence this upregulation correlates with the development of sustained hypersensitivity. Interestingly, this increase was not accompanied by an increase in NR2B protein in the DRG, suggesting that the mRNA increase may be accompanied by an accelerated transport of NR2B protein away from the soma. To investigate this possibility we measured the level of kinesin Kif17. In central neurons Kif17 is involved in the transport into dendrites of various proteins including the NR2B subunit (Setou et al., 2000). Kif17 protein has not been detected in the sciatic nerve (Setou et al., 2000) and primary sensory neurons do not have dendrites. Hence in primary sensory neurons Kif17 most likely is involved in transporting NR2B to the soma’s plasma membrane. Here we found that the expression of Kif17 transcripts was decreased only in sural-SNI. Because of the observed increase in NR2B mRNA, we expect that there should be an increase in NR2B protein production. The observed lack of a change in the measured NR2B protein level within the soma appears to result in part from a decrease in Kif17 which will result in a decrease in NR2B transport to the soma’s plasma membrane. The results also suggest that there may be an increase in axonal transport of NR2B protein away from the soma toward the plasma membrane of fibers and/or nerve terminals and in this way contribute to their hyperexcitability. To our knowledge there is no available information of which kinesin(s) are involved in axonal transport of NR2B, however, we measured the expression of Kif5b since it has been shown to be important in axonal transport of various ion channels and receptors (Rivera et al., 2007; Hirokawa et al., 2010; Twelvetrees et al., 2010; Su et al., 2013). We found that the expression of Kif5b transcripts was not changed. Whether the decrease in Kif17 may promote axonal transport by increasing the availability of NR2B protein to be taken up by axonal transporters, or whether there is an increase in other molecules that mediate axonal transport of NR2B remains to be determined. However, the increase in NR2B mRNA in injured primary sensory neurons correlates with the development of sustained hypersensitivity in sural-SNI.

In sural-SNI, the increase in NR2B mRNA was observed only in L4-DRG, hence enhanced NR2B transcription may be triggered only when a large proportion of sensory fibers are injured (**Table 1**). The hypersensitivity that develops in the rat hind paw from transecting some or all of the sciatic nerve branches is evoked by stimulation of the nerve terminals of the spared sciatic nerve branches (L4-L5 DRG) and from the saphenous nerve (L3-DRG) that become hyperexcitable as a result of interactions with the injured sciatic nerve fibers (Kingery and Vallin, 1989; Kingery et al., 1993; Ro and Jacobs, 1993; Attal et al., 1994; Tal and Bennett, 1994; Guilbaud et al., 1995; Sotgiu and Biella, 1997; Campbell, 2001; Smith et al., 2013). Our results indicate that upregulation of NR2B mRNA may directly contribute to the hypersensitivity of primary sensory neurons located in DRG containing a high number of damaged neurons (L4-DGR) but not to those located in DRG containing a low number of damaged neurons (L3 and L5 DRG).

There was no upregulation of NR2B mRNA in tibial-SNI which correlates with the lack of development of sustained hypersensitivity. Moreover, in tibial-SNI, at Day 86, but not at Day 23 a significant upregulation of NR2B protein was detected within the perinuclear region of the neuronal soma and only in L3-DRG, which contain mostly uninjured saphenous sensory neurons. Uninjured saphenous sensory neurons also become hyperexcitable following injury to the sciatic nerve (Kingery et al., 1993; Ro and Jacobs, 1993; Attal et al., 1994; Guilbaud et al., 1995; Sotgiu and Biella, 1997; Smith et al., 2013). The increase in NR2B protein within the perinuclear region, in the absence of a change in NR2B mRNA, indicates that there is retention and slowing of NR2B transport away from the perinuclear region toward the neuronal plasma membrane. The decrease of NR2B protein transport to the neuronal plasma membrane, would render them non-functional and by doing so would decrease their contribution to neuronal excitability and this may contribute to the recovery of tibial-SNI animals.

In both SNIs, microglia/macrophages showed a transient increase in NR2B protein observed on Day 23 but not on Day 86. This may be related to the reported microglia/macrophage activation following sciatic nerve injuries (Lu and Richardson, 1993; Fenzi et al., 2001; Hu et al., 2007; Vega-Avelaira et al., 2009). Since this transient increase was observed in both SNIs, microglia/macrophage NR2B upregulation may contribute to initial post-injury induced hypersensitivity, but it would appear to not be sufficient for the development of sustained hypersensitivity. At Day 86, but not at Day 23, SGCs displayed an increase in NR2B protein only in tibial-SNI. SGCs become activated following sural-SNI (Xie et al., 2009). Many of the reported injury-evoked alterations in SGCs contribute to neuropathic pain (Hanani et al., 2002; Dublin and Hanani, 2007; Liu et al., 2012); but SGCs could also undergo changes that contribute to recovery. One such alteration could be the increase in NR2B protein since it was observed only in tibial-SNI. How the increase in NR2B expression in SGCs contributes to recovery remains to be determined, but it could involve interactions between SGCs and neurons.

It is known that pain perception shows sex differences (Holdcroft et al., 2000; Ji et al., 2006; Meleine and Matricon, 2014). Moreover, sex hormones have been shown to modulate the somatic sensitivity to nociceptive stimuli (Bradesi et al., 2003; Ji et al., 2003; Sanoja and Cervero, 2005; Nag and Mokha, 2016). This hormonal modulation in part involves regulation of various ion channels and receptors (Lee et al., 2002; McRoberts et al., 2007; Tang et al., 2008; Cho and Chaban, 2012; Ji et al., 2012); including the NMDAr in DRG neurons (McRoberts et al., 2007). Hence additional experiments will be required to determine whether the observations in this study using male rats can be extended to female rats.

In summary, the transient increase of NR2B protein expression in microglia/macrophages correlates with the initial post-injury induced hypersensitivity observed in both SNIs. The increase in NR2B mRNA correlates with the development of sustained hypersensitivity in sural-SNI. The lack of NR2B mRNA increase, together with the retention of NR2B protein within the perinuclear region of the neuronal soma correlates with the recovery from hypersensitivity in Tibial-SNI.

## Author Contributions

MN: Experimental design, surgical procedures, behavioral measurements, tissue collection, tissue processing (for: immunostaining, western blots, and qPCR); immunostaining, western blots, qPCR, data analysis, figure preparation, data discussion, and contributed in writing the manuscript. AS: Assisted in behavioral measurements and surgical procedures; contributed in data discussion and in writing the manuscript. SA and LH: Contributed in immunostaining, data analysis, and data discussion. JZ: Assisted in surgical procedures, behavioral measurements, and data discussion. TB: Contributed in experimental design, data discussion, and manuscript preparation. ER-P: Directed the project, experimental design, discussion, prepared figures, responsible for writing the manuscript.

## Conflict of Interest Statement

The authors declare that the research was conducted in the absence of any commercial or financial relationships that could be construed as a potential conflict of interest.

## Footnotes

1 http://www.stoeltingco.com/media/wysiwyg/58011_Touch_Test_Evaluator.pdf

## Supplementary Material

FIGURE S1 **The paw volume was not affected by Tibial-SNI or Sural-SNI.** The paw volume was measured both in the IL **(A)** and CL **(B)** paws in Tibial-SNI and Sural-SNI rats at the indicated dates. For a given rat the measurement was normalized to the baseline (BL) measurement that was taken the day of the surgery, prior to anesthetizing the animal. No significant difference between groups for a given day. One-way ANOVA (*p* = 0.0055); Bonferroni’s Multiple comparison post-test.

FIGURE S2 **Western blot of NR2B for L3-DRG and L4-DRG. (A)** Western blot for NR2B indicating the NR2B and β-Actin bands of samples from L3-DRG. β-Actin was used to control for the amount of protein loaded. a, naïve IL, b, sham L3 IL, c, Sural-SNI IL, d, Tibial-SNI IL, a’, naïve CL, b’, sham CL, c’, Sural-SNI CL, d’, Tibial-SNI CL. (IL, ipsilateral, CL, contralateral). **(B)** The normalized amount of NR2B protein (NR2B/β-Actin) normalized to the values obtained in sham (control) animals. n: 4 rats No significant difference between each SNI vs. control, or Sural-SNI vs. Tibial-SNI, in either L3-DRG or L4-DRG. One-way ANOVA. **(C)** Low magnification picture (10×) of a DRG section stained with Nissl (green). Scale bar: 50 μm.
