# Supplementary figures and images for: NR2B Expression in Rat DRG Is Differentially Regulated Following Peripheral Nerve Injuries That Lead to Transient or Sustained Stimuli-Evoked Hypersensitivity
Source: Front Mol Neurosci. 2016 Oct 18;9:100. doi: 10.3389/fnmol.2016.00100 (PMC5068091; doi:10.3389/fnmol.2016.00100)

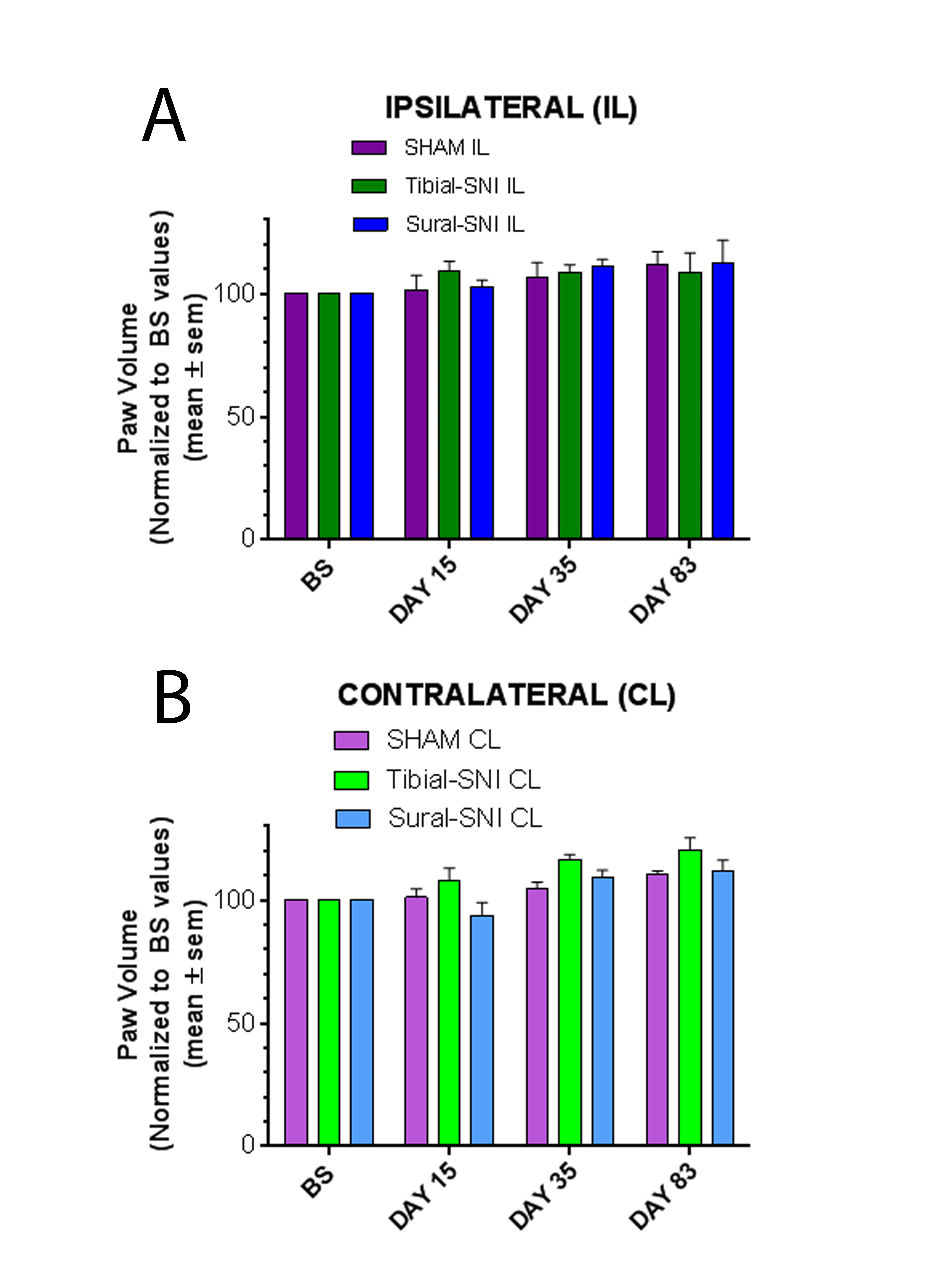

Supplement: FIGURE S1 — The paw volume was not affected by Tibial-SNI or Sural-SNI. The paw volume was measured both in the IL (A) and CL (B) paws in Tibial-SNI and Sural-SNI rats at the indicated dates. For a given rat the measurement was normalized to the baseline (BL) measurement that was taken the day of the surgery, prior to anesthetizing the animal. No significant difference between groups for a given day. One-way ANOVA (p = 0.0055); Bonferroni’s Multiple comparison post-test. [file Image_1.JPEG]

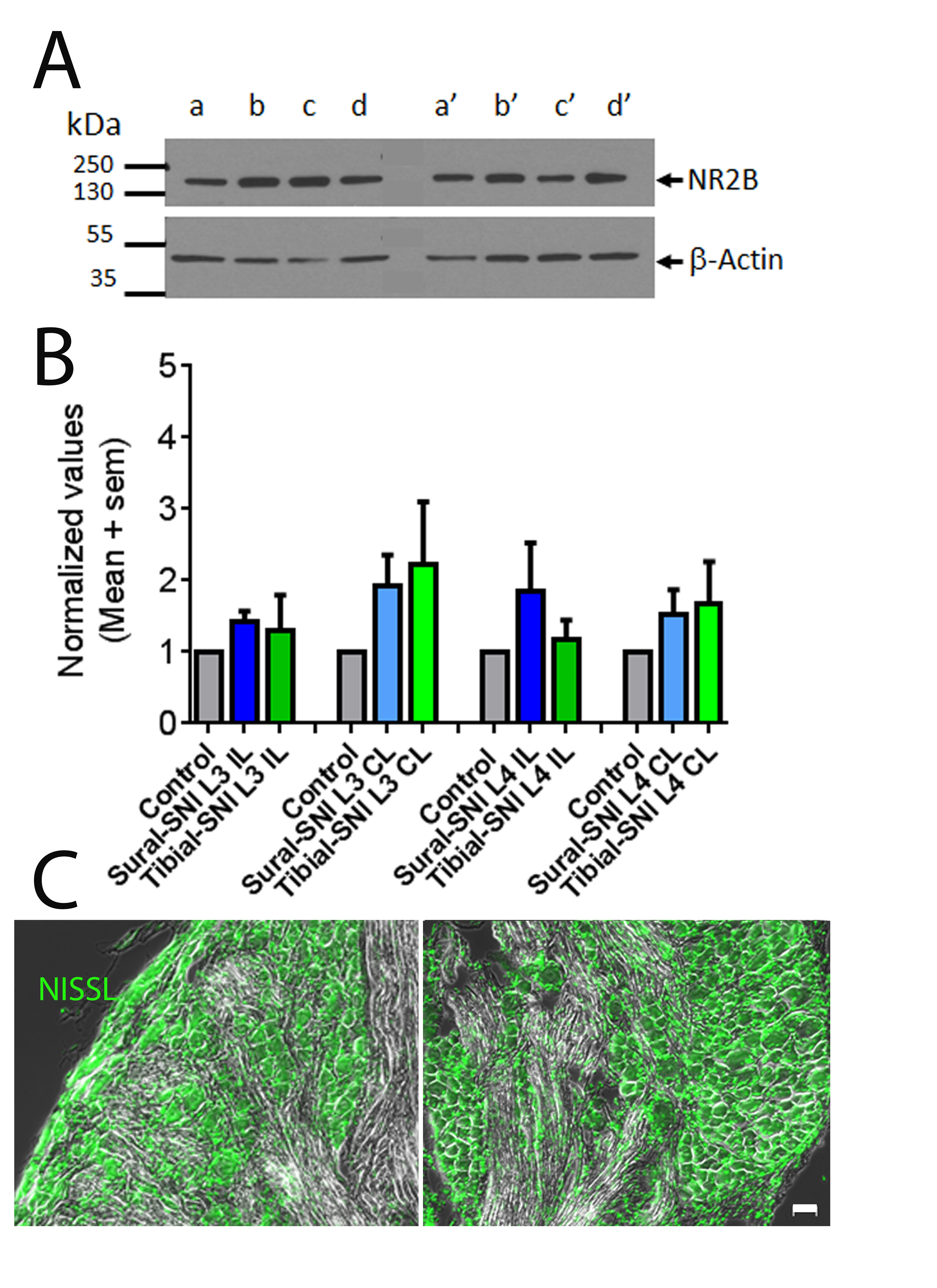

Supplement: FIGURE S2 — Western blot of NR2B for L3-DRG and L4-DRG. (A) Western blot for NR2B indicating the NR2B and β-Actin bands of samples from L3-DRG. β-Actin was used to control for the amount of protein loaded. a, naïve IL, b, sham L3 IL, c, Sural-SNI IL, d, Tibial-SNI IL, a’, naïve CL, b’, sham CL, c’, Sural-SNI CL, d’, Tibial-SNI CL. (IL, ipsilateral, CL, contralateral). (B) The normalized amount of NR2B protein (NR2B/β-Actin) normalized to the values obtained in sham (control) animals. n: 4 rats No significant difference between each SNI vs. control, or Sural-SNI vs. Tibial-SNI, in either L3-DRG or L4-DRG. One-way ANOVA. (C) Low magnification picture (10×) of a DRG section stained with Nissl (green). Scale bar: 50 μm. [file Image_2.JPEG]
